# ^18^F-FET and ^18^F-choline PET-CT in patients with MRI-suspected low-grade gliomas: a pilot study

**DOI:** 10.3325/cmj.2021.62.310

**Published:** 2021-08

**Authors:** Ana Mišir Krpan, Marina Hodolič, Anja Tea Golubić, Maja Baučić, Jakob Nemir, Goran Mrak, Marijan Žuvić, Dražen Huić

**Affiliations:** 1 Department of Oncology, University Hospital Center Zagreb, Zagreb University School of Medicine, Zagreb, Croatia; 2 Nuclear Medicine Research Department, IASON, Graz, Austria; 3 Department of Nuclear Medicine and Radiation Protection, University Hospital Center Zagreb, Zagreb, Croatia; 4 Department of Oncology, University Hospital Center Zagreb, Croatia; 5 Department of Neurosurgery, University Hospital Center Zagreb, Zagreb, Croatia

## Abstract

**Aim:**

To investigate the diagnostic accuracy of O-(2-[^18^F]-fluoroethyl)-L-tyrosine (^18^F-FET) and fluoromethyl-(^18^F)-dimethyl-2-hydroxyethyl-ammonium chloride (^18^F-FCH) computed tomography (CT) in patients with primary low-grade gliomas (LGG).

**Methods:**

The study enrolled patients with magnetic resonance imaging (MRI)-suspected LGG. Patients underwent both ^18^F-FET and ^18^F-FCH positron emission tomography (PET)-CT. Brain PET-CT was performed according to standard protocol – 20 minutes after intravenous injection of 185 MBq of ^18^F-FET and 185 MBq of ^18^F-FCH PET. Surgery and pathohistological diagnosis were performed in the next two weeks.

**Results:**

We observed significantly better concordance between tumor histology and ^18^F-FET PET (weighted Kappa 0.74) compared with both ^18^F-FCH (weighted Kappa 0.15) and MRI (weighted Kappa 0.00). Tumor histology was significantly associated with ^18^F-FET (odds ratio 12.87; 95% confidence interval [CI], 0.49-333.70; *P* = 0.013, logistic regression analysis). Receiver operating characteristic curve analysis comparing ^18^F-FCH (area under the curve [AUC] 0.625, 95% CI 0.298-0.884) and ^18^F-FET (AUC 0.833, 95% CI 0.499-0.982) showed better diagnostic properties of ^18^F-FET (AUC difference 0.208, 95% CI -0.145 to 0.562, *P* = 0.248).

**Conclusion:**

Performing PET-CT in patients with newly diagnosed LGG should be preceded by a selection of an appropriate radiopharmaceutical. ^18^F-FET seems to be more accurate than ^18^F-FCH in the LGG diagnosis.

Low-grade gliomas (LGG) have been receiving increasing attention due to a better understanding of their natural history and clinical diversity, improvements in pathological classification, development of diagnostic and treatment modalities, and new clinical trials. All of this has changed the management paradigm. LGG are a group of relatively uncommon, diffusely infiltrative malignancies (ie, astrocytomas, oligodendrogliomas) classified as grade II according to the World Health Organization (WHO) 2016 grading system. These gliomas account for approximately 15% of all gliomas, with the incidence rate of 1/100 000 persons per year (1). They arise mostly in the younger and middle-age group, with an average age at diagnosis of 35 years. Although traditionally considered benign, LGG gradually evolve into high-grade tumors. This happens in approximately half of the patients within five years (2). Since LGG are potentially curable tumors, patients need to be correctly diagnosed and treated according to the guidelines. The main treatment modalities are surgery, radiation therapy, and chemotherapy. Patients with low-risk tumors and an indolent disease course, in whom long survival is expected, require active surveillance.

The diagnosis of gliomas is based on clinical symptoms, clinical history, brain imaging, and histological and molecular data. Combining different radiological and nuclear medicine techniques allows the visualization of different morphological and functional changes.

Preoperative imaging of brain lesions patients with gliomas enables the assessment of size, location, relationship to the functional centers and white matter tracts, as well as of tumor grade. In this setting, multiparametric magnetic resonance imaging (MRI), including both conventional and functional sequences, such as diffusion-weighted imaging (DWI), perfusion-weighted imaging (PWI), and MR spectroscopy (MRS) serves as a gold standard. LGG usually present as hypointensities on T1-weighted imaging (T1W) and hyperintensities on T2-weighted imaging (T2W) and fluid attenuated inversion recovery (FLAIR) imaging. The lesions can present with intratumoral calcifications or cysts and minimal perilesional edema. Contrast enhancement, if present, is minimal. Nevertheless, although contrast enhancement has been associated with a higher degree of malignancy, some degree of contrast enhancement may be seen in up to 60% of LGG (3). In particular, LGG are characterized by less restricted diffusion on diffusion-weighted sequences, and perfusion parameters similar or lower than that of normal white matter. MRS can help to clarify the diagnosis but does not make it definitive. MRS findings are non-specific, indicative of the neoplastic nature, but not of the degree. A more accurate diagnosis of patients with LGG could be provided by an integration of morphological and functional imaging modalities.

Diagnostic possibilities and therapeutic strategies in patients with brain tumors are enhanced by the development of hybrid technology as well as the availability of specific radiopharmaceuticals. Positron emission tomography-computed tomography (PET-CT) scanning is one of the most promising modalities in this setting.

O-(2-[^18^F]-fluoroethyl)-L-tyrosine (^18^F-FET) has been approved as a PET radiopharmaceutical for the characterization of brain lesions suggestive of gliomas. ^18^F-FET accumulates in glioma cells due to an increased expression of L-amino acid transporters LAT 1, LAT 2, and LAT 3. ^18^F-FET has the advantage of displaying a high tumor-to-background ratio and of not accumulating in inflammatory lesions. Several studies have indicated that ^18^F-FET PET in combination with MRI can improve the diagnostic and therapeutic assessment of patients with gliomas for neurosurgery (4-6).

Radioactive choline is a tracer of choice in PET imaging of patients with prostate cancer. The use of choline in PET imaging is based on increased phosphorylcholine levels and an elevated phosphatidylcholine turnover in malignant cells. The use of radioactive choline in brain tumors was first described in 1997 (7,8). Because of low uptake in normal brain parenchyma, fluoromethyl-(^18^F)-dimethyl-2-hydroxyethyl-ammonium chloride (^18^F-FCH) is still a good alternative in diagnostic centers where ^18^F-FET is not available.

A rat model comparing ^18^F-FET and ^18^F-choline showed a better performance of ^18^F-FET in gliomas (9). No study so far has compared ^18^F-FCH and ^18^F-FET in patients with LGG. The aim of this pilot study was to determine the diagnostic accuracy of ^18^F-FET and ^18^F-FCH in patients with LGG.

## Methods

This prospective single-center study enrolled 11 patients treated at University Hospital Center Zagreb, Croatia, from February 2018 until June 2019. The inclusion criteria were age ≥18 years and MRI-suspected LGG reviewed by experienced neuro-radiologists as newly diagnosed supratentorial tumors that are amenable for resection or biopsy in patients with Karnofsky score ≥80. The study was approved by the Ethics Committee of Zagreb University Hospital Center (8.1-17/47-2, 02/21 AG). All patients signed informed consent before any study-related procedure. Histological results were compared with MRI, ^18^F-FCH PET-CT, and ^18^F-FET PET-CT findings.

### Magnetic resonance imaging protocol

MRI examinations were performed on a 3T MRI machine (Siemens Prisma Trio, Siemens AG, Munich, Germany) equipped with a 64-channel head coil. Each patient was scanned with a sagittal 3D FLAIR sequence (TR = 5000 ms, TE = 397 ms, TI = 1800 ms, acquired voxel size 1.0 × 1.0 × 1.0 mm) and a sagittal 3D T1-weighted sequence (TR = 2300 ms, TE = 3 ms, TI = 900 ms, flip angle = 9°, acquired voxel size 1.0 × 1.0 × 1.0 mm). All patients underwent diffusion tensor imaging and tractography (Neuro 3D, Siemens). The specific parameters were as follows: TR = 2680 ms, TE = 48 ms, FOV = 280 mm, matrix size = 128 × 128, number of signal averages = 1, slice thickness = 4.0 mm, b = 0 and 500 s/mm^2^, and the scanning time was 6 minutes and 49 seconds. Pre-surgical blood oxygen level-dependent functional MRI (fMRI) was performed using an echo planar imaging/gradient echo protocol (TR = 3000 ms, TE = 30 ms, slices number = 38, slice thickness = 3.5 mm, FOV = 280, matrix size = 128 × 128, voxel dimension = 3.5 × 3.5 × 3.5 mm, flip angle = 90, bandwidth = 4808 Hz/Px).

### ^18^F-FET and ^18^F-FCH PET-CT protocol

Patients underwent both ^18^F-FET and ^18^F-FCH PET-CT scanning within one week. PET-CT brain imaging was performed (Siemens Biograph mCT, Siemens Medical Solutions USA, Inc, Malvern, PA, USA,) according to a standard protocol (20 minutes static image, 4 frames each for 5 minutes) 20 minutes after an intravenous injection of 185 MBq of ^18^F-FET (IASOGlio, IASON, Graz-Seiersberg, Austria) and 185 MBq ^18^F-FCH (IASOcholine, IASON). Standardized uptake value (SUV) was calculated.

### Histology

Tissue was processed according to the standard procedure. Tissue samples were formalin-fixed paraffin-embedded, and stained with hematoxylin and eosin. Isocitrate dehydrogenase 1 (IDH1 R132H) mutation was determined by immunohistochemistry. IDH2 and O(6)-methylguanine-DNA methyltransferase status was not assessed. Histological diagnosis was obtained independently and by consensus of two expert neuropathologists from the same institution. Tumors were classified according to the 2016 World Health Organization Classification of Tumours of the Central Nervous System (1).

### Surgery

Six patients underwent awake craniotomy and five patients underwent craniotomy under general anesthesia. All patients were presurgically examined and prepared by a specialized team consisting of a neurosurgeon, neurologist, speech therapist, and neuroanesthesiologist according to awake craniotomy standards. In all patients, preoperative 3T MR brain imaging with fMRI and tractography and preoperative PET-CT scan were performed.

We operated on all patients in a “asleep-awake-asleep” modality. Patients were positioned mostly in a lateral decubital position or in a position according to the tumor site. During the procedure, a neuronavigation system, intraoperative ultrasound, and intraoperative brain mapping were used. Brain mapping was started after the patient was fully awake and adequately responsive. After brain mapping, transcortical concept approach was performed for tumor removal. The goal was supramarginal resection every time if it was feasible according to brain mapping results. The tumor was removed with cavitron ultrasonographic surgical aspirator. Control MRI was performed within 48 hours and three months after surgical procedure.

### Statistical analysis

The sample size was not calculated because of the initial limited sample size and the preliminary study design. Categorical data are presented as prevalence, and continuous variables as median and range. The concordance of MRI and ^18^F-FET and ^18^F-FCH PET-CT with histological diagnosis was assessed with the Kappa test. The association of ^18^F-FET and ^18^F-FCH PET-CT data and other independent variables with a positive histology was assessed with logistic regression analysis. Diagnostic properties were evaluated, and ^18^F-FET and ^18^F-FCH PET-CT were compared using receiver operating characteristic (ROC) curve analysis. All tests were two sided, and *P* < 0.05 was considered to be significant. Statistical analysis was performed with MedCalc Statistical Software, version 19.1 (MedCalc Software bv, Ostend, Belgium).

## Results

The mean age was 42 years (range 21-80 years). There were six female patients. Tumors were located as follows: 6 in the frontal lobes, 3 in the temporal lobes, 1 in the parietooccipital lobe, and 1 in the insula. All patients had an MRI-suspected LGG. In all PET-positive patients, tumor location on MRI was consistent with the region of PET-CT positivity.

Nine patients had positive 18F-FET PET findings, and two out of these nine had additional 18F-FCH PET/CT-positive findings.

Two patients with an MRI-suspected LGG did not undergo surgery and histological confirmation. Both had a negative ^18^F-FET PET and a negative ^18^F-FCH PET-CT scan, so they refused operation at the moment, and the multidisciplinary tumor board suggested a close follow-up. Nine patients with a suspected LGG underwent full imaging diagnostics with final histological findings after surgery.

**Figure 1:**
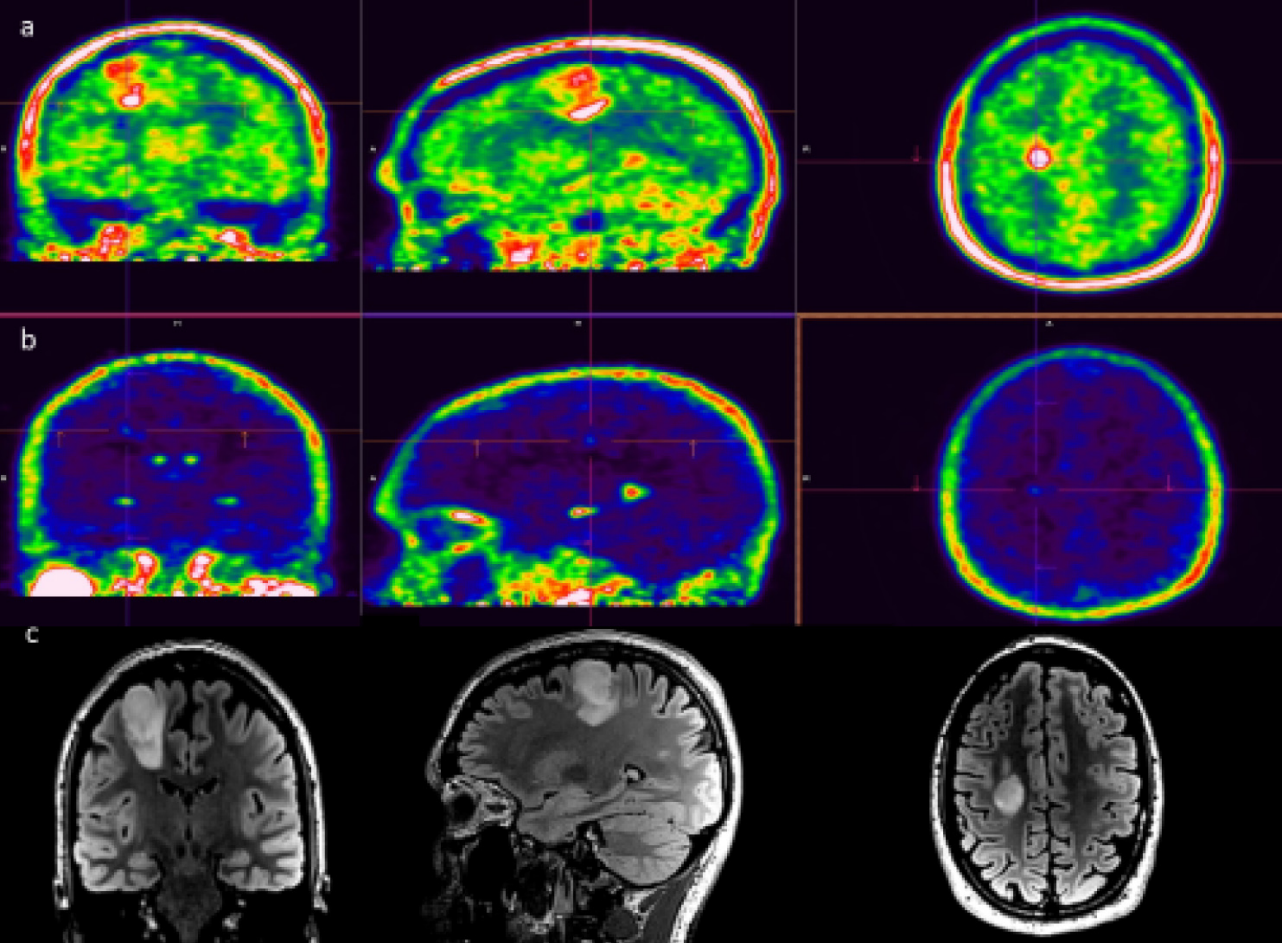
Positron emission tomography-computed tomography (PET-CT) and magnetic resonance imaging (MRI) fluid attenuated inversion recovery (FLAIR) images of a 38-year-old male patient with an isocitrate dehydrogenase 1-mutated 1p/19q-non-codeleted World Health Organization grade II diffuse astrocytoma. O-(2-[^18^F]-fluoroethyl)-L-tyrosine PET was positive (standardized uptake value [SUV] max 2.8) (**A**) and fluoromethyl-(^18^F)-dimethyl-2-hydroxyethyl-ammonium chloride PET was minimal (SUV max 0.47) (**B**). MRI FLAIR tumor in the right frontal lobe (**C**).

**Figure 2:**
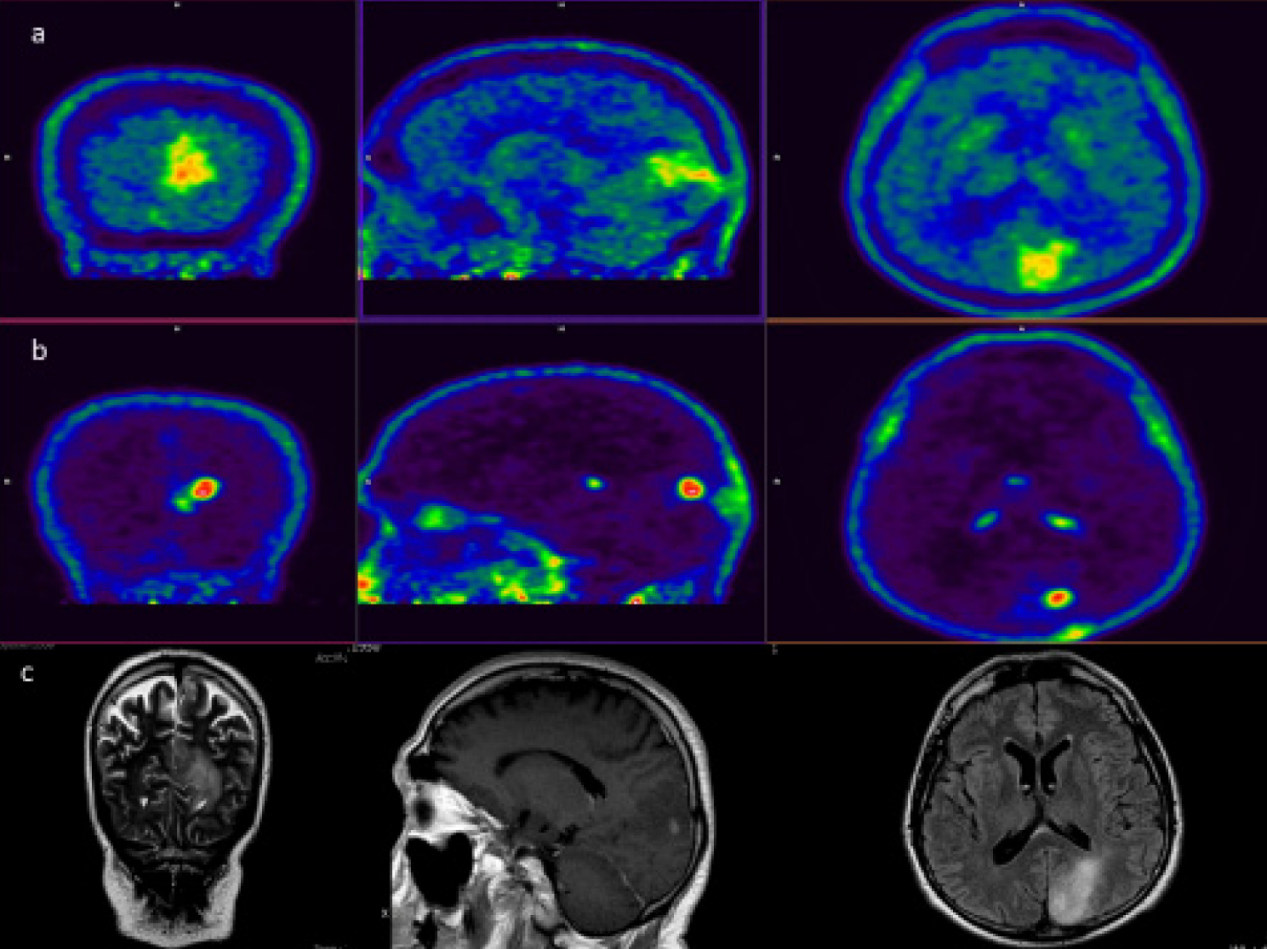
Positron emission tomography-computed tomography (PET-CT) and magnetic resonance imaging (MRI) fluid attenuated inversion recovery (FLAIR) images of a 69-year-old male patient with an isocitrate dehydrogenase 1-wildtype glioblastoma. Both   O-(2-[^18^F]-fluoroethyl)-L-tyrosine PET (**A**) and fluoromethyl-(^18^F)-dimethyl-2-hydroxyethyl-ammonium chloride PET-CT (**B**) were positive (standardized uptake value max 3.9 and 3.1, respectively). MRI FLAIR tumor in the left parietooccipital region (**C**).

Six patients were histologically diagnosed as LGG according to the WHO 2016 classification, grade I or grade II: all were positive on ^18^F-FET PET (SUVmax: 1.7, 2.0, 2.8, 1.8, 1.5, and 1.7) and negative on ^18^F-FCH PET-CT scan (Figure 1). One lesion had a minimal/nonsignificant ^18^F-FCH uptake (SUVmax 0.47) so we defined it as negative. Three patients were confirmed as having a high-grade glioma. Two patients were diagnosed with glioblastoma. Both of them were positive on both tracers: ^18^F-FCH PET-CT (SUVmax 3.9 and SUVmax 1.6) and ^18^F-FET (SUVmax 3.1 and SUVmax 3.0) (Figure 2). One patient had anaplastic astrocytoma grade III, ^18^F-FCH negative and ^18^F-FET PET positive (SUVmax 1.3) (Table 1).

**Table 1:** The results of the diagnostic workup of patients with an magnetic resonance imaging-suspected low-grade glioma (N = 11)*

Patient number	FCH PET	FCH SUV max	FET PET	FET SUV max	Histology	Grade	IDH status
1	negative	0	positive	1.7	diffuse astrocytoma	II	NOS
2	negative	0	negative	0.0	NA	NA	NA
3	negative	0	positive	2.0	diffuse astrocytoma	II	mutant
4	positive	1.6	positive	3.0	glioblastoma	IV	mutant
5	negative	0	positive	2.8	diffuse astrocytoma	II	mutant
6	positive	3.9	positive	3.1	glioblastoma	IV	NOS
7	negative	0	positive	1.8	ganglioglioma	I	NA
8	negative	0	positive	1.5	diffuse astrocytoma	II	mutant
9	negative	0	negative	0.0	NA	NA	NA
10	negative	0	positive	1.3	anaplastic astrocytoma	III	mutant
11	negative	0	positive	1.7	ganglioglioma	I	NA

*Abbreviations: NA – not applicable; NOS – not otherwise specified; FET PET – O-(2-[^18^F]-fluoroethyl)-L-tyrosine positron emission tomography; FCH PET – fluoromethyl-(^18^F)-dimethyl-2-hydroxyethyl-ammonium chloride; IDH – isocitrate dehydrogenase; SUV – standardized uptake value.

**Figure 3:**
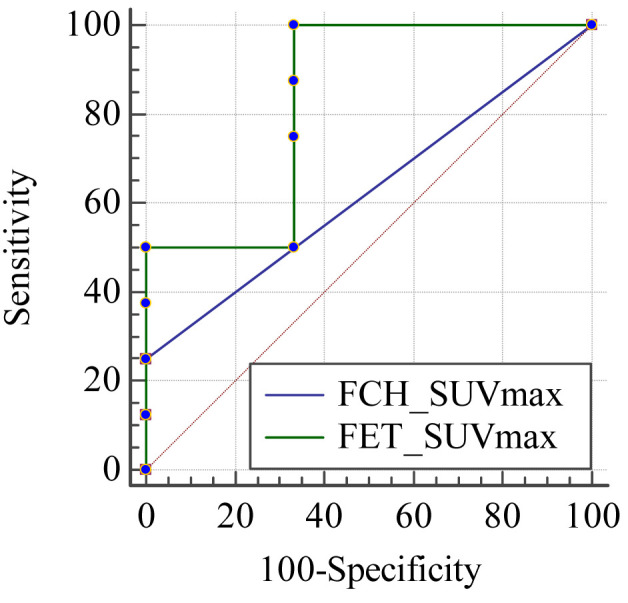
Receiver operating characteristic curves for the prediction of sensitivity and specificity of fluoromethyl-(^18^F)-dimethyl-2-hydroxyethyl-ammonium chloride maximum standardized uptake value and  O-(2-[^18^F]-fluoroethyl)-L-tyrosine maximum standardized uptake value.

Significantly better concordance was found between tumor histology and ^18^F-FET PET (weighted Kappa 0.74) compared with both ^18^F-FCH (weighted Kappa 0.15) and MRI (weighted Kappa 0.00). Tumor histology was significantly associated with ^18^F-FET (odds ratio 12.87; 95% confidence interval [CI], 0.49-333.70; *P* = 0.013, logistic regression analysis). Although the result was not significant (possibly due to a small sample), ROC curve analysis comparing ^18^F-FCH (area under the curve [AUC] 0.625, 95% CI 0.298-0.884) and ^18^F-FET (AUC 0.833, 95% CI 0.499-0.982) showed better diagnostic properties of ^18^F-FET (AUC difference 0.208, 95% CI -0.145 to 0.562, *P* = 0.248) (Figure 3).

The correlation with IDH status was not investigated because of a small sample size. Out of eleven patients, only five had IDH1 mutation. IDH2 testing is not performed in our laboratory, and two patients had no histological diagnosis.

## Discussion

The current study found significantly better concordance between tumor histology and ^18^F-FET PET compared with both 18F-FCH and MRI. Until recently the treatment algorithm for LGGs has been controversial. These tumors mainly occur in younger patients, who under proper treatment can achieve long progression-free and overall survival. Since these patients have a good prognosis, it is very important to keep in mind the late treatment effects and carefully plan the treatment from the beginning. Due to the wide availability of brain MRI, suspected lesions are detected even in asymptomatic patients. Findings of the brain MRI help the neurosurgeon to decide between surgery and the wait-and-watch approach. Therefore, non-invasive preoperative glioma diagnosis needs to be accurate and precise.

A meta-analysis of MRI accuracy for LGG diagnosis (10) showed the AUC of T2-weighted imaging, MR spectroscopy, and T2/FLAIR-weighted imaging to be 89.0%, 78.1%, and 77.4%, respectively. The diagnostic accuracy of T2-weighted imaging was 89.0%, 0.4%, 44.7%, and 205 measured as AUC, false-positive rate, true-positive rate, and diagnostic odds ratio, respectively (10). If LGG is suspected on MRI, the state-of-the-art approach is to proceed with upfront surgical treatment aimed at maximizing surgical resection and obtaining the tissue for further analysis. A wait-and-watch approach could be detrimental for the patient if the tumor progresses quickly. Obtaining a glioma tissue sample still remains mandatory since the disease course can be predicted only from molecular markers, which in the era of molecular profiling and targeted drugs can have a crucial effect on the treatment.

To increase the diagnostic accuracy, the diagnostic algorithm for LGG started to include nuclear medicine methods, as well as new methods in the field of neuro-radiology, for example 2-hydroxyglutarate-proton magnetic resonance spectroscopy or MRI perfusion (11-13).

In our small patient series, we noticed insufficient accuracy of preoperative brain MRI. Of the 11 patients, high-grade glioma was histologically confirmed in three patients. This means that in these patients delaying surgery based on only brain MRI would lead to a rapid clinical disease progression. Furthermore, two patients were histologically diagnosed with a benign disorder of focal cortical dysplasia and glioma grade I. In similar situations, surgery is performed only exceptionally. Other four patients who underwent surgery had histological findings of LGGs grade II consistent with brain MRI.

Choline plays a role in the synthesis of phospholipid components of the cell membrane. It is phosphorylated by choline kinase to phosphocholine, and then metabolized to phosphatidylcholine. Once phosphorylated, phosphocholine is trapped within the cell. Choline kinase and choline transporters overexpression is a common feature of several malignancies, including gliomas. Radiolabeled choline is routinely used in daily management of patients with prostate and hepatocellular cancer. Its low uptake in the normal brain potentially provides good contrast with brain lesions (14,15). Owing to the mentioned reasons and market availability, radiolabeled choline is used in the management of patients with gliomas in many nuclear medicine departments in Europe. Two radioactive cholines, ^11^C-choline and ^18^F-FCH, were demonstrated to have a similar uptake in gliomas (16).

^18^F-FET is an ideal tracer for brain tumor assessment due to its high *in vivo* stability, high tumor-to-background contrast, and tissue specific tracer kinetics. The uptake of ^18^F-FET is mediated by system L-amino acid transporters. The dependency of ^18^F-FET uptake on the breakdown of the blood brain barrier (BBB) was found to be lower than that of radio-labeled choline (17,18). However, some preclinical studies found similar and even higher BBB dependency of ^18^F-FET compared with choline (15,19). Only one study evaluated the use of ^18^F-FCH PET in LGG, including six patients without previous treatment (20).

The current study demonstrated that ^18^F-FET PET detected LGG infiltration more accurately than ^18^F-FCH PET. Five patients with histologically proven LGG displayed clear ^18^F-FET and no ^18^F-FCH uptake on PET. In these patients, SUVmax varied between 1.5 and 2.8. Glioblastoma presented with pathological ^18^F-FET but also with ^18^F-FCH uptake. Intense ^18^F-FCH uptake in high-grade gliomas (HGG) was reported in cell-line studies (21). In our study, SUVmax (up to 3.1) was significantly higher in glioblastomas than in LGG.

In four of our patients, ^18^F-FET uptake was clearly more diffuse and corresponded to MRI-described lesions, while ^18^F-FCH uptake was more intense, focal, and visible only in parts of ^18^F-FET and MRI lesions in these patients.

Difference in ^18^F-FET and ^18^F-FCH uptake in gliomas can be explained by disrupted BBB in HGG (transport mechanism of choline is different from that of FET) or by the relation of ^18^F-FCH uptake to capillary density, not just disrupted BBB (15).

Despite having promising results, our study has some limitations. The number of patients is relatively small. PET was performed 20 minutes after ^18^F-FET injection. A recently published study from Amsterdam suggested that ^18^F-FET/PET 60-90-min interval might have a higher diagnostic accuracy than the 20-40-min interval (22). Other authors have also reported better detection of diffuse glioma at intervals longer than 60 min compared with shorter intervals (23). In our study, PET imaging sequence included a CT scout topogram, followed by a static single field of view acquisition of PET images, as proposed by Law et al (11). In the current study, only static single field of view PET images were acquired due to the small sample size and comparable results. An established clinical value of dynamic PET images acquisition is applicable only to ^18^F-FET studies (24).

A meta-analysis by Treglia et al (25) discussed other PET-CT studies with different tracers for brain tumors. The authors reported several common limitations, mainly due to the sample size or tracer availability. Heterogeneity of tumors also plays an important role in functional imaging and reporting. Different acquisition modalities were reported. In a pooled results analysis of suspicious brain tumors, of the four radiotracers evaluated (^18^F-FDOPA, ^18^F-FET, ^11^C-methionin, and ^18^F-FDG), ^18^F-FET PET-CT had a high sensitivity confidence interval, higher than ^18^F-FDG.

Our results strongly support the need to complement structural MRI with nuclear medicine procedures in patients with gliomas. The practical implication of our study is that daily management of patients with gliomas should include the use of an appropriate PET radiopharmaceutical. Our study suggests that if ^18^F-FCH is the only available tracer, it can still be used to confirm or exclude higher tumor malignancy. If both tracers are available, ^18^F-FET should be the first choice for tumor characterization before therapy.

Our results showed that performing PET-CT scan in patients with MRI-suspected low-grade gliomas should be preceded by a selection of an appropriate radiopharmaceutical. The ^18^F-FET PET-CT more accurately detects LGG than ^18^F-FCH. Both tracers seem to be appropriate in primary diagnosis of high-grade gliomas.
